# Whole-Cell Screen of Fragment Library Identifies Gut Microbiota Metabolite Indole Propionic Acid as Antitubercular

**DOI:** 10.1128/AAC.01571-17

**Published:** 2018-02-23

**Authors:** Dereje A. Negatu, Joe J. J. Liu, Matthew Zimmerman, Firat Kaya, Véronique Dartois, Courtney C. Aldrich, Martin Gengenbacher, Thomas Dick

**Affiliations:** a Antibacterial Drug Discovery Laboratory, Department of Microbiology and Immunology, Yong Loo Lin School of Medicine, National University of Singapore, Singapore; b Tuberculosis Research Laboratory, Department of Medicine, Yong Loo Lin School of Medicine, National University of Singapore, Singapore; c St. Peter TB Specialized Hospital, Addis Ababa, Ethiopia; d Public Health Research Institute, New Jersey Medical School, Rutgers, The State University of New Jersey, Newark, New Jersey, USA; e Department of Medicinal Chemistry, College of Pharmacy, University of Minnesota, Minneapolis, Minnesota, USA

**Keywords:** tuberculosis, fragments, indole propionic acid, gut microbiota

## Abstract

Several key antituberculosis drugs, including pyrazinamide, with a molecular mass of 123.1 g/mol, are smaller than the usual drug-like molecules. Current drug discovery efforts focus on the screening of larger compounds with molecular masses centered around 400 to 500 g/mol. Fragment (molecular mass < 300 g/mol) libraries have not been systematically explored for antitubercular activity. Here we screened a collection of 1,000 fragments, present in the Maybridge Ro3 library, for whole-cell activity against Mycobacterium tuberculosis. Twenty-nine primary hits showed dose-dependent growth inhibition equal to or better than that of pyrazinamide. The most potent hit, indole propionic acid [IPA; 3-(1*H*-indol-3-yl)propanoic acid], a metabolite produced by the gut microbiota, was profiled *in vivo*. The molecule was well tolerated in mice and showed adequate pharmacokinetic properties. In a mouse model of acute M. tuberculosis infection, IPA reduced the bacterial load in the spleen 7-fold. Our results suggest that IPA should be evaluated as an add-on to current regimens and that fragment libraries should be further explored to identify antimycobacterial lead candidates.

## INTRODUCTION

Tuberculosis (TB) remains a global health threat, killing 1.34 million people in 2016 (1). The high prevalence of drug-resistant Mycobacterium tuberculosis strains is a medical urgency and calls for the development of new drugs active against TB (2, 3). Some of the key antituberculosis drugs, discovered by whole-cell or animal model screening, such as pyrazinamide (PZA), are dirty fragments (4). They hit multiple targets in different pathways, and their molecular masses are in the range of 100 to 300 g/mol. This type of mechanism of action, polypharmacology, and their physicochemical properties, very small and reactive, are at odds with mainstream antibacterial drug discovery. In addition, several fragment-like anti-TB drugs are activated inside M. tuberculosis to generate reactive and promiscuous metabolites. For medicinal chemists, however, attractive leads should inhibit a single target to facilitate lead optimization, be large enough to bind a target with a high affinity, and not be reactive to minimize side effects (4).

We recently argued that the success of small dirty drugs in tuberculosis chemotherapy suggests that fragment-based whole-cell screens should be reintroduced into our current antimycobacterial drug discovery efforts (4, 5). The physicochemical properties of fragments, small and moderately lipophilic, may be useful for achieving *in vivo* exposure and tissue penetration (6). In the case of mycobacteria, these physicochemical properties likely also have a positive impact on bacterial cell penetration: fragments might more easily penetrate the double-membrane mycobacterial cell envelope since porins, the channels spanning the outer membrane, prefer small hydrophilic molecules (7, 8). Thus, fragments may have multiple advantages over larger molecules: favorable absorption and systemic pharmacokinetic (PK) properties, favorable tissue distribution, and better bacterial uptake (4).

Here we carried out such a screen of a collection of fragment-sized compounds typically used for structure-based lead discovery (9) for their activity against M. tuberculosis. We identified a series of whole-cell active hits and tested the most potent compound in the mouse.

## RESULTS

### Whole-cell screen and hit confirmation.

**FIG 1:**
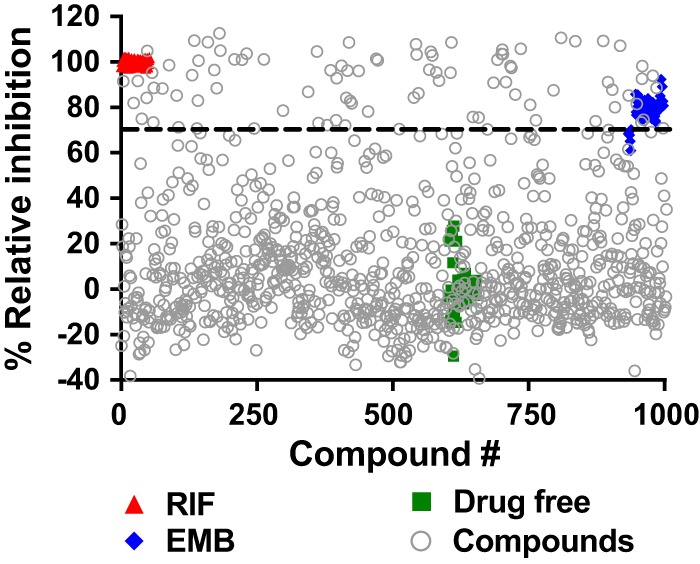
Whole-cell screening of the activities of the fragments against M. tuberculosis. A scatter plot of relative M. tuberculosis growth inhibition in the primary screen performed in a 96-well format is shown. Fragment compounds (1 mM) were incubated with exponentially growing M. tuberculosis bacteria prior to cell density measurement at 600 nm. The growth inhibition in the wells with the fragments was calculated relative to the growth in drug-free control wells. Rifampin and ethambutol were used at concentrations of 10 μM and 6 μM, respectively. The data points shown are the averages of two biological replicates. Standard deviations were <20%.

**FIG 2:**
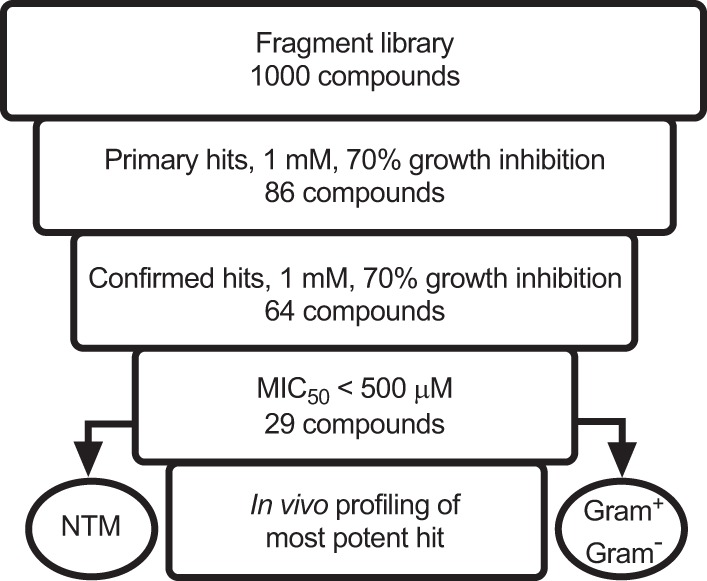
Compound progression flowchart.

To screen the Maybridge library of fragment-like molecules for growth inhibition, we employed a simple and robust M. tuberculosis screening assay in a 96-well format using turbidity measurement as the readout. A primary single-point screen was performed with two biological replicates. Compounds that showed at least 70% growth inhibition were defined as hits (Fig. 1). From 86 primary hits, 64 could be confirmed by the same single-point screen using reordered solids (Fig. 2). These confirmed hits were subjected to M. tuberculosis growth inhibition dose-response assays, resulting in 29 whole-cell-active compounds with MIC_50_ (MIC inhibiting 50% of growth) of <500 μM (Fig. 2; Table 1). This cutoff was chosen on the basis of the MIC_50_ of the fragment-like first-line drug pyrazinamide (10).

**TABLE 1:** Antibacterial and cytotoxicity profiles of fragment hits^*a*^

Fragment	IUPAC name	Bactericidal activity (log reduction) at 1 mM	Activity (MIC_50_ [μM]) in acidic medium (pH 6.2)	Antibacterial activity (MIC_50_ [μM])							Cytotoxicity screening					
				M. tuberculosis	M. bovis BCG	M. smegmatis	M. avium	M. abscessus	S. aureus	E. coli	THP-1 cells		HepG2 cells		RBCs	
											CC_50_ (μM)	SI	CC_50_ (μM)	SI	HC_50_ (μM)	SI
F1	3-(1*H*-Indol-3-yl)propanoic acid	1.76	62	68	98	477	119	>500	>500	>500	>1,000	>14.7	>1,000	>14.7	>1,000	>14.7
F2	7-Hydroxy-4-(trifluoromethyl)-2*H*-chromen-2-one	1.10	179	213	329	476	>500	>500	198	432	879	4.1	987	4.6	>1,000	>4.7
F3	2-Methyl-1*H*-imidazole-4-carbothioamide	1.87	98	105	134	>500	198	304	>500	>500	850	8.1	>1,000	>9.5	>1,000	>9.5
F4	Methyl isoquinoline-3-carboxylate	1.98	158	175	143	389	344	>500	>500	>500	>1,000	>5.71	>1,000	>5.71	>1,000	>5.71
F5	6,7,8,9-Tetrahydrodibenzo [*b*,*d*]furan-2-amine	1.11	62	245	488	>500	>500	>500	>500	>500	>1,000	>4.1	>1,000	>4.1	>1,000	>4.1
F6	9*H*-Pyrido[3,4-*b*]indole	0.43	113	97	205	121	302	254	>500	302	230	2.4	476	4.9	787	12.5
F7	6-Methyl-4-piperazino-2-(trifluoromethyl)quinoline	3.02	144	101	246	345	267	423	>500	302	478	4.7	345	3.4	>1,000	>14.9
F8	5-(4-Chlorophenyl)-*N*,*N*,2-trimethyl-3-furamide	1.44	93	135	189	233	169	322	>500	>500	234	1.7	589	4.4	433	3.2
F9	Methyl 3-hydroxy-1-benzo thiophene-2-carboxylate	2.09	240	142	>500	>500	>500	>500	217	>500	657	4.6	544	3.8	>1,000	>7.0
F10	5-Phenylthiophene-2-carboxylic acid	1.46	97	186	397	345	>500	247	>500	>500	544	2.9	612	3.3	>1,000	>5.4
F11	3-(4-Fluorophenyl)-5-(methylsulfanyl)-1*H*-pyrazole	3.06	140	195	308	398	498	271	>500	344	765	3.9	945	4.8	>1,000	>5.1
F12	6-Chloro-2-(1,4-diazepan-1-yl)-1,3-benzothiazole	2.80	104	198	137	221	157	351	>500	324	124	0.6	187	0.9	>1,000	>5.1
F13	2-(3-Chlorophenoxy) ethanethioamide	0.90	75	211	>500	409	387	289	>500	>500	656	3.1	467	2.2	904	4.3
F14	Isoquinoline-3-carboxylic acid	1.97	87	235	193	176	473	>500	304	193	523	2.2	678	2.9	>1,000	>4.2
F15	4-(4-Chlorophenoxy)-3,5-dimethyl-1*H*-pyrazole	2.43	157	235	156	198	174	256	79	>500	345	1.5	676	2.9	453	1.9
F16	2,5-Dimethyl-1-(2-thienylmethyl)-1*H*-pyrrole-3-carboxylic acid	1.89	345	247	>500	>500	>500	>500	>500	>500	724	2.9	766	3.1	>1,000	>4.0
F17	[6-(Piperidin-1-yl)pyridin-2-yl]methanamine	3.07	245	250	345	>500	271	>500	104	>500	489	2.0	455	1.8	>1,000	>4.0
F18	2-(2,2,4,7-Tetramethyl-1,2,3,4-tetrahydroquinolin-1-yl)ethan-1-ol hydrate	1.67	86	268	87	205	198	247	189	>500	246	0.9	198	0.7	471	1.8
F19	Ethyl 2-amino-5-methyl-4-phenylthiophene-3-carboxylate	1.98	117	270	267	491	214	206	344	>500	247	0.9	233	0.9	453	1.7
F20	[2,2′-Bithiophene]-5-carboxylic acid	1.98	170	271	457	289	>500	267	>500	>500	974	3.6	765	2.8	>1,000	>3.7
F21	2-Methyl-1*H*-indol-5-amine	2.50	305	281	398	401	>500	>500	487	>500	409	1.5	387	1.4	>1,000	>3.6
F22	2-Fluoro-4-hydroxybenzonitrile	1.60	140	300	420	487	317	401	343	202	677	2.3	879	2.9	>1,000	>3.3
F23	Methyl[(2-phenoxyphenyl) methyl]amine	1.22	>500	305	347	>500	>500	>500	>500	>500	976	3.2	789	2.6	>1,000	>3.3
F24	2-Methyl-5-(4-methylphenyl)-3-furoic acid	2.02	120	316	193	>500	458	>500	>500	>500	887	2.8	984	3.1	>1,000	>3.2
F25	5-Chloro-1-benzothiophene-3-carboxylic acid	2.10	94	319	367	98	489	257	>500	>500	876	2.7	765	2.4	945	3.0
F26	4-(3-Thienyl)benzoic acid	1.35	125	320	>500	>500	>500	497	>500	>500	978	3.1	944	3.0	>1,000	>3.1
F27	6-Chlorobenzo[*d*]isoxazol-3-ol	1.05	210	395	>500	>500	>500	>500	>500	>500	777	2.0	>1,000	>2.5	>1,000	>2.5
F28	{2-[4-(Trifluoromethyl)phenyl]-1,3-thiazol-4-yl}methanol	1.21	215	434	398	235	325	253	>500	>500	940	2.2	>1,000	>2.3	786	1.8
F29	4-Phenoxyphenol	1.09	118	439	387	250	411	267	348	201	145	0.3	202	0.5	912	2.1

a THP-1, monocytic cell line; HepG2, liver cell line; RBC, red blood cells; CC_50_ and HC_50_, cytotoxic and hemolytic concentrations that killed or lysed 50% of cells relative to the number of untreated control cells, respectively; SI, selectivity index, calculated as CC_50_/MIC_50_ or HC_50_/MIC_50_. Bactericidal activity indicates activity against M. tuberculosis.

### Bactericidal activity determination.

The bactericidal activity of the 29 most potent hits was evaluated by treatment of M. tuberculosis cultures with the compounds at 2 times the cutoff concentration and subsequent CFU enumeration on agar. We observed that half of the whole-cell-active compounds produced a 100-fold reduction in viability, and three hits produced a 1,000-fold reduction in viability (Table 1).

### Determination of activity spectrum.

**FIG 3:**
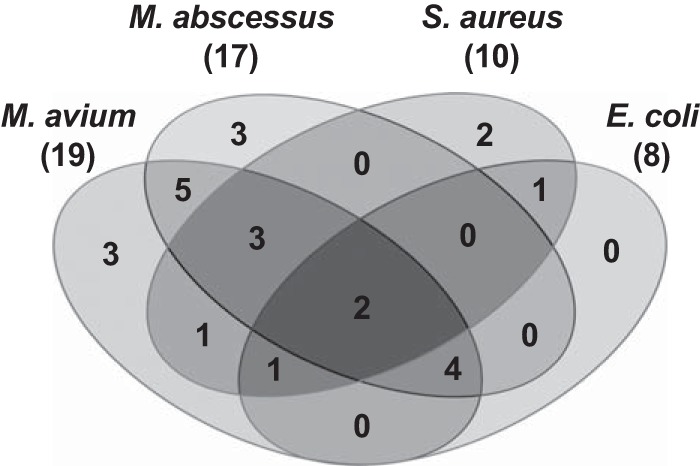
Activity spectrum of 29 fragment hits with anti-M. tuberculosis activity. The Venn diagram shows the overlapping activities (MIC_50_ < 500 μM) of the M. tuberculosis-specific hits with two nontuberculous mycobacteria (M. avium and M. abscessus), S. aureus, and E. coli.

Next we assessed the spectrum of activity of the 29 compounds active against M. tuberculosis against the nontuberculous mycobacterial (NTM) pathogens M. abscessus and M. avium (11), as well as Staphylococcus aureus and Escherichia coli (Table 1). A large fraction of fragments active against M. tuberculosis showed activity against the two NTM species (Fig. 3). Less overlapping activity was detected for the representatives of Gram-positive and Gram-negative bacteria (Fig. 3).

### Cytotoxicity and hemolysis determination.

The cytotoxicity of the 29 hits for cells of two human cell lines, HepG2 and THP-1, was measured. Membrane toxicity was assessed by a red blood cell lysis assay. Several of the compounds active against M. tuberculosis displayed acceptable cytotoxicity and hemolytic activity with a selectivity index of 5 or above (Table 1).

### *In vivo* profiling of the most potent hit.

**FIG 4:**
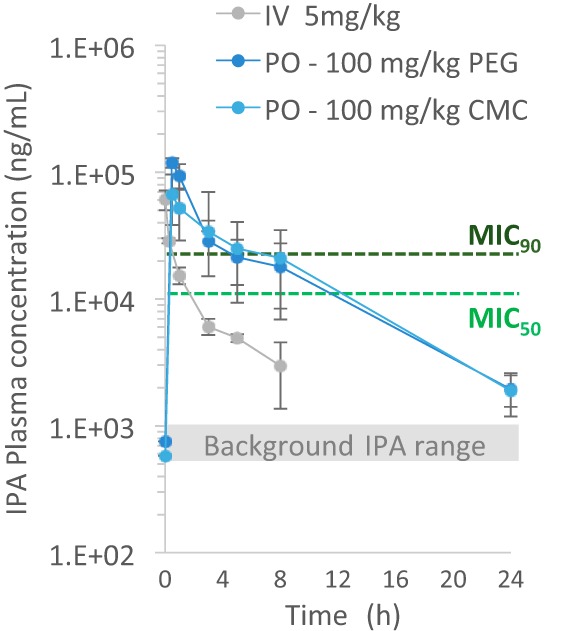
Concentration-time profile of indole propionic acid (IPA) in plasma following intravenous (IV) and oral (PO) administration, as indicated. The range of IPA concentrations found in control mice is indicated by the gray window. CMC, carboxymethyl cellulose-based formulation (suspension); PEG, polyethylene glycol-based formulation (solution). The MIC_50_ and MIC_90_ are indicated by dashed lines.

**FIG 5:**
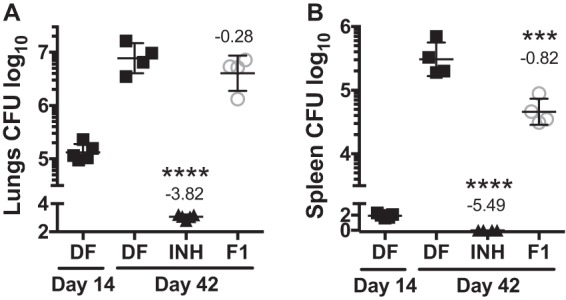
Evaluation of indole propionic acid (IPA) in mice with acute M. tuberculosis infection. At 14 days postinfection with aerosolized M. tuberculosis, chemotherapy was initiated for 4 weeks. IPA (100 mg/kg) and INH (25 mg/kg) were given on 6 days/week by oral gavage. The bacterial burden in the lungs (A) and spleen (B) at designated time points was determined by plating organ homogenates on agar, followed by incubation and colony counting. Data obtained from two independent studies were analyzed by a one-way analysis of variance multicomparison with the Bonferroni posttest (*n* = 4 or 5 mice). ***, *P* < 0.001; ****, *P* < 0.0001. Shown are data points for individual mice, presented as means and SDs. Numbers above the groups reflect mean differences from the values for the drug-free (DF) control at day 42.

The most attractive hit based on M. tuberculosis whole-cell activity and *in vitro* tolerability [fragment F1, or 3-(1*H*-indol-3-yl)propanoic acid, also known as indole propionic acid (IPA; molecular mass, 189 g/mol; logarithm of partition coefficient between *n*-octanol and water, 2.15; hydrogen bond acceptor, 2; hydrogen bond donor, 2; polar surface area, 53 Å (12)] was selected for acute toxicity testing in mice. Three animals were dosed at 100 mg/kg of body weight on three consecutive days and monitored for 7 days after receiving the last dose. None of the mice showed abnormal behavior over the course of the experiment or gross pathological changes of major organs after termination of the study. *In vivo* pharmacokinetic profiling of IPA revealed adequate exposure relative to potency when 100 mg/kg was delivered via the oral route (Fig. 4) and 30 to 33% oral bioavailability (see Table S1 in the supplemental material). Plasma levels were above the MIC_50_ for more than 50% of the dosing interval. The plasma of control mice that did not receive IPA contained approximately 300 to 1,000 ng/ml of endogenous IPA (see Discussion). Average concentrations and the associated standard deviations (SDs) measured throughout the day in 9 naive mice were 629 ± 233 ng/ml (Table S2). To evaluate whether *in vitro* activity would translate into *in vivo* efficacy, we employed the mouse model of acute tuberculosis. Mice were infected with a low dose of M. tuberculosis by the aerosol route. At 14 days postchallenge, the bacterial burden in the lungs had reached 10^5^ CFU, indicating establishment of an acute pulmonary infection (Fig. 5). At this time, disease began to disseminate from the lungs to secondary organs, such as the spleen, where up to 100 bacilli/animal were detected (Fig. 5). Chemotherapy was initiated at 14 days postinfection, and the drug formulations were administered at 100 mg/kg on six consecutive days per week for 4 weeks. None of the animals showed signs of adverse events or abnormal behavior over the course of drug treatment, confirming that IPA is well tolerated. The first-line drug isoniazid (INH) was used as a control; it reduced the bacterial load of the lungs and sterilized the spleen, as expected, within 4 weeks of monotherapy (Fig. 5) (13). At the end of the experiment, mice that received IPA had a 7-fold lower bacterial load in the spleen than untreated mice (Fig. 5). In conclusion, we demonstrate that one fragment hit, IPA, displayed *in vivo* tolerability, attractive pharmacokinetics, and activity.

## DISCUSSION

Current antituberculosis drug discovery efforts focus on the screening of libraries of drug-like molecules, the majority of which have a molecular mass centered around 500 g/mol. Libraries of fragment-sized compounds have not been systematically interrogated for antitubercular activity (4, 5). Here we screened a collection of fragments for their activity against M. tuberculosis and identified 29 molecules that showed MIC_50_s comparable to or lower than the MIC_50_ of the first-line fragment drug pyrazinamide (10). *In vivo* profiling of the most potent hit, indole propionic acid [IPA; 3-(1*H*-indol-3-yl)propanoic acid], showed that it has attractive tolerability, adequate pharmacokinetic properties, and efficacy in a mouse model of tuberculosis. Interestingly, IPA lowered the number of CFU only in the spleen. The reasons for the apparent organ-specific effect of IPA remain to be determined. Possible explanations include unequal tissue penetration (6), a differential immune response in spleen and lungs, and/or the differential, organ-specific susceptibility of bacilli to IPA as a response to the specific microenvironments experienced by the pathogen (14, 15). It is interesting to note that IPA has been reported to show neuroprotective, antioxidant, and antiamyloid properties (16) and that the compound (SHP-622, VP-20629) is in early clinical development for the treatment of the progressive neurodegenerative disease Friedreich's ataxia (https://clinicaltrials.gov/ct2/show/NCT01898884). Intriguingly, IPA is a metabolite produced endogenously by the gut microbiota and can be detected in the blood of the host (17). Accordingly, we found 0.5 to 1 μg/ml of IPA in the plasma of untreated mice (Fig. 4; see Table S2 in the supplemental material). Recently, Dodd et al. (18) identified the genes in the gut bacterium Clostridium sporogenes that encode the production of IPA. Furthermore, the authors identified Peptostreptococcus anaerobius and three strains of Clostridium cadaveris to be additional IPA-producing gut bacteria. Importantly, it was shown that IPA has effects on intestinal permeability and the innate arm as well as the adaptive arm of the immune system (12, 18, 19). The *in vitro* and *in vivo* antitubercular activity of the compound demonstrated here may suggest an effect of the gut microbiota on tuberculosis disease susceptibility, progression, and/or severity (20, 21). To what extent the observed *in vivo* activity of IPA is due to its direct antibacterial activity and its host immune-modulatory function remains to be determined.

Taken together, we screened a library of 1,000 fragment-like molecules for whole-cell activity against M. tuberculosis and found that one unoptimized fragment hit, IPA, displayed *in vivo* tolerability and exposure, as well as activity in a mouse model of tuberculosis. Our results suggest that fragment libraries should be further explored as a source of chemical starting points. IPA should be evaluated as an add-on to current regimens and as a starting point to deliver more potent analogues. Studies are in progress to characterize the *in vitro* and *in vivo* antibacterial mechanism of action of this fragment compound and to determine *in vivo* synergies with clinically used antituberculosis drugs.

## MATERIALS AND METHODS

### Animals and ethics assurance.

Mouse studies were carried out in accordance with the *Guide for the Care and Use of Laboratory Animals* of the National Institutes of Health (22) with approval from the Institutional Animal Care and Use Committee of the New Jersey Medical School, Newark, NJ (CD-1 mice), and the National University of Singapore's Institutional Animal Care and Use Committee (BALB/c mice). All animals were maintained under specific-pathogen-free conditions and fed water and chow *ad libitum*, and all efforts were made to minimize suffering or discomfort. Studies in M. tuberculosis-infected animals were performed in biosafety level 3 facilities approved for the containment of M. tuberculosis.

### Chemicals and drugs.

The Maybridge Ro3 library, consisting of 1,000 chemically diversified fragment compounds in liquid format, was purchased from Maybridge USA. Rifampin (RIF), PZA, INH, ethambutol (EMB), and dimethyl sulfoxide (DMSO) were procured from Sigma-Aldrich, USA. All compounds were dissolved in 90% DMSO at a 10 mM concentration and stored in aliquots at −80°C until use.

### Screening.

Primary screening was performed using exponentially growing M. tuberculosis H37Rv (ATCC 27294) at an optical density at 600 nm (OD_600_) of ∼0.4 and a final drug concentration of 1 mM in 96-well flat-bottom clear microtiter plates (Corning). The inoculum was adjusted to a final OD_600_ of 0.05 in Middlebrook 7H9 (Becton, Dickinson) broth medium supplemented with 0.05% Tween 80, 0.2% glycerol, and 10% albumin-dextrose-catalase enrichment. A suspension of 200 μl of inoculum was seeded into the prepinned 96-well plates containing 1 μl of 200 mM fragment. RIF (10 μM) and EMB (6 μM) were included in each microtiter plate as positive controls with high and medium potencies, respectively. The plates were sealed with a Breathe-Easy membrane (Diversified Biotech) and incubated for 4 days at 37°C and 80 rpm orbital shaking. The cell density at 600 nm was determined using a Tecan Infinite 200 Pro microplate reader, and percent growth inhibition in reference to the growth in a drug-free control containing 0.5% DMSO was calculated. Compounds causing >70% growth inhibition in two biological replicates were defined as primary hits.

### Inhibitory and bactericidal activity.

The MICs reducing bacterial growth by 50% or 90% were determined as previously described (23). Briefly, drug concentrations ranging from 1 to 1,000 μM were used to inhibit the growth of exponentially growing M. tuberculosis H37Rv in 96-well plates. The absorbance at 600 nm was measured after 10 days of incubation at 37°C and 80 rpm orbital shaking. Bactericidal activity was determined by subculturing bacilli treated with 1,000 μM the respective compounds on Middlebrook 7H11 agar supplemented with 10% oleic acid-albumin-dextrose-catalase enrichment and 0.5% glycerol. Agar plates were incubated for 3 to 4 weeks at 37°C prior to counting. The absolute number of log CFU per milliliter reduction was calculated by subtracting the remaining number of log CFU per milliliter of treated bacilli from the initial inoculum.

### Spectrum of activity.

Exponentially growing M. bovis BCG Pasteur (ATCC 335734), M. smegmatis (ATCC 700084), M. avium (ATCC 35717), M. abscessus (ATCC 19977), Staphylococcus aureus (ATCC 12600), and Escherichia coli (ATCC 25922) were used as assay strains. For M. bovis BCG, the experimental setup described for M. tuberculosis was used. Cultures of M. smegmatis, M. avium, and M. abscessus were diluted in Middlebrook 7H9 medium to a final OD of 0.005 and incubated with the test compound in clear 96-well flat-bottom microtiter plates for 1 day, 4 days, and 3 days, respectively. Culture of S. aureus and E. coli were diluted in LB medium and incubated with the test compound overnight. Ten 2-fold serial dilutions of compounds starting from a 1 mM concentration were used (5, 23).

### Cytotoxicity.

Cells of the human hepatocyte cell line HepG2 (ATCC HB-8065) and the human monocytic cell line THP-1 (ATCC TIB-202) were maintained in 5% humidified CO_2_ and at 37°C in Dulbecco's modified Eagle medium and RPMI 1640 medium, respectively. The media were supplemented with 10% heat-inactivated fetal calf serum and 2 mM glutamine (both from Gibco). THP-1 cells were differentiated using 40 ng/ml phorbol 12-myristate 13-acetate overnight (24).

The cytotoxicity assay was performed as described previously (25). Briefly, 20,000 HepG2 cells or 60,000 THP-1 cells were seeded into wells of 96-well plates and incubated overnight to allow cell adherence. Cells were exposed to test compounds at concentrations ranging from 62.5 to 1,000 μM for 24 h. Cell viability was assessed by use of a CellTiter 96 AQueous One Solution cell proliferation kit following the manufacturer's instruction (Promega). The hemolytic concentration of the compounds that lysed 50% of cells (HC_50_) was determined by exposing human red blood cells (RBCs; Interstate Blood Bank, Inc., Laboratory, USA) to drugs at concentrations ranging from 62.5 to 1,000 μM. The cytotoxic concentrations that killed 50% of cells (CC_50_) were calculated by a nonlinear logistic model equation using GraphPad Prism (version 6) software. Selectivity index (SI) values were defined by the ratio of CC_50_/MIC_50_ or HC_50_/MIC_50_.

### PK analyses.

Pharmacokinetic (PK) studies were performed in uninfected CD-1 mice after administration of single doses of 3-(1*H*-indol-3-yl)propanoic acid (IPA) at 5 mg/kg via the intravenous (i.v.) route and 100 mg/kg via the oral (p.o.) route as described previously (26). The i.v. formulation was 5% dimethylacetamide (DMA)–95% of a 4% Cremophor solution. The p.o. formulation was either 50% polyethylene glycol (PEG) 400–50% dextrose 5% in sterile water (D5W) to generate a solution or 0.5% carboxymethyl cellulose (CMC) and 0.5% Tween 80 in water to generate a suspension. In the i.v. arm, blood was collected in K_2_EDTA-coated tubes predose and at 1 min, 15 min, and 1, 3, 5, 8, and 24 h postdose. In the p.o. arms, blood was collected predose and at 15 min, 30 min, and 1, 3, 5, 8, and 24 h postdose. Plasma was obtained by centrifugation for 10 min at 5,000 rpm and stored at −80°C until analyzed. IPA concentrations were measured as described below. The lower limit of quantification was 5 ng/ml. The PK parameters (area under the curve [AUC] from time zero to time *t* [AUC_0–*t*_] and area under the curve from time zero to 24 h [AUC_0–24_], peak [maximum] plasma concentration [*C*_max_], and half-life [*t*_1/2_]) were calculated from the mean concentrations using Microsoft Excel software (Office 2010; Microsoft Corp., Redmond, WA). AUCs were calculated using the linear trapezoidal rule. Half-life and elimination rate constants were calculated by linear regression using semilogarithmic concentration-versus-time data.

### Analytical methods.

Neat 1-mg/ml DMSO stocks of IPA were first serially diluted in 50/50 acetonitrile-water and subsequently serially diluted in drug-free CD-1 mouse plasma (K_2_EDTA; Bioreclamation IVT, NY) to create standard curves and quality control (QC) spiking solutions. Twenty microliters of standards, QC samples, control plasma, and study samples was extracted by adding 200 μl of acetonitrile-methanol (50/50) protein precipitation solvent containing the internal standard (10 ng/ml verapamil). Extracts were vortexed for 5 min and centrifuged at 4,000 rpm for 5 min. One hundred microliters of supernatant was transferred for high-pressure liquid chromatography coupled to tandem mass spectrometry (LC/MS-MS) analysis and diluted with 100 μl of Milli-Q-deionized water.

LC/MS-MS quantitative analysis for IPA was performed on a AB Sciex Qtrap 6500+ triple-quadrupole mass spectrometer coupled to a Shimadzu 30ACMP high-pressure liquid chromatography system, and chromatography was performed on an Agilent Zorbax SB-C8 column (2.1 by 30 mm; particle size, 3.5 μm) using a reverse-phase gradient elution. Milli-Q-deionized water with 0.1% formic acid was used for the aqueous mobile phase, and 0.1% formic acid in acetonitrile was used for the organic mobile phase. Multiple-reaction monitoring (MRM) of parent/daughter transitions in the electrospray positive-ionization mode was used to quantify all molecules. MRM transitions of 190.10/130.10 and 455.40/165.20 were used for IPA and the internal standard, respectively. Sample analysis was accepted if the concentrations of the quality control samples and standards were within 20% of the nominal concentration. Data processing was performed using Analyst software (version 1.6.2; Applied Biosystems Sciex).

### Animal tolerability and efficacy experiments.

Eight- to 10-week-old female BALB/c mice were maintained in groups of 3 or 4 in individually ventilated cages under specific-pathogen-free conditions at the National University of Singapore biosafety level 3 core facility. Food and water were offered *ad libitum*. Test drugs were formulated in equal volumes of polyethylene glycol 400 and 5% glucose and administered at a dose of 100 mg/kg in a volume of 200 μl by oral gavage. Acute toxicity was assessed by dosing groups of 3 mice on three consecutive days followed by a monitoring period of 7 days. Animals were subsequently euthanized by CO_2_ to assess gross pathological changes. For determination of the *in vivo* efficacy of drug candidates, mice were infected with 100 to 200 CFU M. tuberculosis H37Rv using a full-body inhalation exposure system (GlasCol). After 14 days, chemotherapy was initiated at 6 days per week for 4 weeks. INH at a dose of 25 mg/kg formulated in 0.25% methyl cellulose served as a control. Mice were euthanized at designated time points by CO_2_. The bacterial burden of the organs was determined by plating serial dilutions of organ homogenates onto Middlebrook 7H11 agar supplemented with 20 μg/ml ampicillin and 10 μg/ml cycloheximide. Colonies were counted after 3 to 4 weeks of incubation at 37°C.

## Supplementary Material

Supplemental material
