# Supplementary material for: Whole-Cell Screen of Fragment Library Identifies Gut Microbiota Metabolite Indole Propionic Acid as Antitubercular
Source: Antimicrob Agents Chemother. 2018 Feb 23;62(3):e01571-17. doi: 10.1128/AAC.01571-17 (PMC5826148; doi:10.1128/AAC.01571-17)
Supplement: Supplemental material [file supp_62_3_e01571-17__index.html]

Supplemental material 

# Whole-Cell Screen of Fragment Library Identifies Gut Microbiota Metabolite Indole Propionic Acid as Antitubercular

## Supplemental material

- Supplemental file 1 -

  Supplemental Tables S1 and S2

  PDF, 343K
