# Supplementary material for: Whole-Cell Screen of Fragment Library Identifies Gut Microbiota Metabolite Indole Propionic Acid as Antitubercular
Source: Antimicrob Agents Chemother. 2018 Feb 23;62(3):e01571-17. doi: 10.1128/AAC.01571-17 (PMC5826148; doi:10.1128/AAC.01571-17)
Supplement: Supplemental material [file AAC.01571-17_zac002186910s1.pdf]

Supplemental material

Table S1. Pharmacokinetic parameters of indole propionic acid (IPA) following intravenous and oral administration to CD-1 mice.

Route/dose/formulation	$AUC_{[0-8]}$ (µg*h/mL) Ave +/- SD (n=3)	$AUC_{[0-24]}$ (µg*h/mL) Ave +/- SD (n=3)	C_{max} (µg/mL) Ave +/- SD (n=3)	α-$T_{1/2}$ (h)	V_d (L/kg)	F (%)
Intravenous 5 mg/kg	72 +/- 3	n.d.	n.a.	1.27	0.25	n.a.
Oral 100 mg/kg in CMC	315 +/- 88	442 +/- 271	67 +/- 28	n.a.	n.a.	30.8
Oral 100 mg/kg in PEG	312 +/- 59	471 +/- 129	118 +/- 10	n.a.	n.a.	32.9

AUC: area under the concentration-time curve; C_{max}: peak plasma concentration; α-$T_{1/2}$: rate of decline in plasma concentrations due to drug distribution from the central to the peripheral compartment; V_d: volume of distribution; F: oral bioavailability. CMC: carboxymethylcellulose based suspension; PEG: polyethyleneglycol based solution. The β-$T_{1/2}$ or elimination half-life could not be calculated due to the presence of endogenous IPA.

Table S2. Endogenous plasma levels (ng/mL) of indole propionic acid (IPA) in naïve mice (non-TB infected and untreated) measured at different times during the day

Time (h)	Mouse 1	Mouse 2	Mouse 3	Mouse 4	Mouse 5	Mouse 6	Mouse 7	Mouse 8	Mouse 9	**Average**	SD*
0.25	503	538	726	486	759	402	920	430	509	**589**	120
1	439	459	645	374	584	443	739	373	463	**514**	114
3	373	359	550	420	618	380	899	600	539	**455**	106
5	477	301	433	389	713	502	622	605	456	**404**	92
8	883	425	578	715	843	959	726	775	888	**629**	233

*Standard Deviation
